# Adherence to the Lamartina-Berjano Classification and Suggested Surgical Treatment Decreases the Rate of Postoperative Mechanical Failures in Adult Deformity Patients. A Retrospective Observational Study With a Minimum 10 Years Follow-Up

**DOI:** 10.1177/21925682251332555

**Published:** 2025-03-28

**Authors:** D. Compagnone, L. La Verde, A. Redaelli, D. Solano, F. Langella, M. Damilano, D. Vanni, C. Lamartina, P. Berjano, R. Cecchinato

**Affiliations:** 1 46767 IRCCS Ospedale Galeazzi, Milan, Italy; 2 University of Milan, Milan, Italy; 3 Center for Research and Training in Neurosurgery (CIEN), Hospital Universitario de la Samaritana, Bogotà, Colombia; 4 Universidad Del Rosario, School of Medicine and Health Sciences, Bogotà, Colombia; 5 Department of Biomedical Sciences for Health, University of Milan, Milan, Italy

**Keywords:** sagittal alignment, sagittal balance, deformity

## Abstract

**Study Design:**

Retrospective cohort analysis.

**Objectives:**

To evaluate the effectiveness of the Lamartina-Berjano (L-B) classification in reducing mechanical complications in patients with adult spinal deformities, with a minimum follow-up of 10 years.

**Methods:**

The study included cases of adult deformity with at least 10 years of follow-up. The rate of clinically-relevant mechanical complications, defined as any implant-related issue requiring revision surgery, was estimated. The independent variable was adherence to the treatment guidelines of the L-B classification. The analysis was limited to patients with thoracolumbar deformities, and the population was stratified according to postoperative alignment using GAP scores.

**Results:**

A total of 121 patients met the inclusion and exclusion criteria. In this cohort, the revision surgery rate for clinically-relevant mechanical complications was 49.6% (60 out of 121 patients). Of these, 90 patients (74%) had surgery following the L-B classification guidelines. A lower risk of complications was observed in aligned patients whose surgeries adhered to the L-B classification. Additionally, the survival curve showed significant differences between patients who followed L-B guidelines and those who did not.

**Conclusion:**

Our retrospective analysis shows that following the L-B classification guidelines leads to a reduction in mechanical complications in patients with thoracolumbar deformities, particularly in a long-term follow-up scenario.

## Introduction

Spinal deformity in adults is a spectrum of pathologies that lead to an alteration of the spinal alignment,^1^ with a reported prevalence up to 32% in general population and higher in elderly people,^2^ frequently causing an increase in axial pain and a reduction of quality of life and function.^3^ The surgical correction of these deformities often requires complex and challenging surgeries, with long fusions of the spine and eventual osteotomies to adequately correct the spinal shape. Unfortunately, in this population of patients, the rate of intraoperative and postoperative complications is definitively high, with a readmission rate of these patients that reaches 56% in specific studies.^4^ It has been demonstrated that unplanned re-operations in this set of patients is associated with a reduced quality of life at a 5-year follow-up.^5^ In the recent years scientific literature tried to evaluate and propose different methods and classification systems, as SRS-Schwab classification^6^ and Lenke classification extension for adult deformity,^7^ with the aim of reducing these complications, especially when considering mechanical failures. One of these classifications has been proposed in 2014 by Lamartina and Berjano.^8^ It divides the spinal deformity patients in seven categories, based on the location of the deformity and on compensating mechanisms, and provides an indication on the surgical treatment for each group.

Aim of this retrospective observational study is to verify the efficacy of this classification in reducing the rate of mechanical complications in a 10-year follow-up scenario.

## Materials and Methods

### Study Design and Patients

Retrospective cohort analysis of cases of adult deformity with sagittal imbalance correction in a high-volume division (*GSpine 4*, *IRCCS Ospedale Galeazzi - Sant’Ambrogio*, *Milan*, *Italy*), with 10 years minimum follow-up.

### Inclusion and Exclusion Criteria

Patients older than 18 affected by adult spinal deformity with sagittal imbalance and an indication for surgical correction were included in the study. They must have been treated before the proposal of the Lamartina and Berjano classification, in order to reduce the selection bias, with a posterior instrumented fusion of at least 4 vertebrae, and a minimum follow-up of 10 years with complete clinical or radiological data. To reduce the variability of the outcomes on possible biases in results, we decided to limit the analysis to patients with thoracolumbar deformities, thus belonging to the following groups of the classification: Thoracic Hyperkyphosis, Thoracolumbar Kyphosis, Lumbar Kyphosis, Lower Lumbar Kyphosis, Global Kyphosis.

Patients were excluded from analysis if they had received other previous spine fusion procedures, in case of diagnosis of neuromuscular diseases, rheumatic diseases, active infections or tumors, or in case of unavailability of pre-operative, early post-operative (between 0 and 12 weeks) and at least 10 years after the surgery clinical and radiological data.

Sagittal Imbalance was defined as the presence of at least one of the following radiological parameters before surgery: Sagittal Vertical Axis (SVA) > 5 cm. Thoracic Kyphosis (TK) > 60°. Thoraco-lumbar Junction (TLJ) Kyphosis >10°. Pelvic Tilt (PT) ≥ 20°.

## Study Variables

### Cohort Descriptive Variable

#### Demographic and Intraoperative Variables

Age at the time of surgery and sex were the demographic variables collected before the surgical procedure. Data on surgical techniques and number of instrumented levels were also collected.

#### Radiographic Variables

In all enrolled patients we measured pelvic incidence (PI), pelvic tilt (PT), sacral slope (SS), maximum lumbar lordosis (LL), lordosis between L4 and S1 (LLL), thoracic kyphosis (TK), thoraco-lumbar junction (TLJ) kyphosis, the sagittal vertical axis (SVA) and Global Tilt (GT). We classified all the deformities according to Lamartina-Berjano Classification preoperatively and evaluated the GAP-score^9^ in the early postoperative.

#### Mechanical Complication

We estimated the rate of clinically-relevant mechanical complication, defined as any mechanical complication related to spinal implant that needed revision surgery.

We categorized clinically-relevant mechanical complications into complications related to the implant (implant failures–IF) (breakage of the rods, loosening, pull-out or breakage of screws or interbody cages, non-union), and junctional failures (JF), divided in proximal junctional failure (PJF), defined by the occurrence of upper instrumented vertebra (UIV) or UIV +1 fracture, pull-out of UIV instrumentation, presence of sagittal subluxation or symptomatic proximal junctional kyphosis; distal junctional failure (DJF), when lower instrumented vertebra (LIV) or LIV +1 fracture or pull-out of the LIV instrumentation occurred.

#### Predictor Variables

We considered as independent variable the respect of treatment indication by Lamartina-Berjano Classification. The population was stratified according to postoperative alignment, based on GAP score values. A score from 0 to 4 indicates a proportioned balance, while a score >4 describes a disproportionate spinopelvic balance. The cut-off value was selected according to a Receiver Operator Characteristic (ROC) curve that showed a GAP score threshold value that optimized the relationship between sensitivity and specificity (higher Youden’s index = [sensitivity - (1-specificity)]) = 4 (Youden’s Index = 1,37).

### Data Collection

Eligible patients were identified from the Institute’s clinical database, containing surgical reports, from 2008 to 2014. All patients were enrolled in accordance with inclusion and exclusion criteria. All the surgical procedures performed for each patient were reviewed. All patients were evaluated on an outpatient basis at the last follow-up to provide information on any complication or subsequent spinal surgery performed in our or other hospitals. For each surgery, radiographic variables were collected for pre-operative, immediate post-operative, obtained within the first 12 weeks, and at the last available FU or before revision surgery. The radiographic measurements were performed using a properly designed and validated software (Surgimap Spine Software, Nemaris Inc, New York, USA).^10^ Patients with radiographic examinations that did not meet the protocol criteria were excluded from the study.^11^ All measurements were performed in the same hospital by two of the authors (L.V.L. and S.D.). Preoperative classification according to Lamartina-Berjano was performed by two of the authors and all disagreements were resolved by a meeting held in consultation with a third author (C.D.). All data regarding the surgical revisions due to mechanical failure were recorded. Mechanical failure was assessed as independent outcomes of the study.

### Statistical Analysis

Data were analyzed using SPSS version 21.0 (SPSS Inc, Chicago, IL, USA). Descriptive statistics were used to present demographics and radiographic parameters. Categorical data were presented with absolute frequencies and percentages; Continuous variables were expressed as mean ± standard deviation.

For the statistical association between surgical revision for mechanical failure and the respect of Lamartina-Berjano Classification in the treatment, a tables 2x2 were built (Respect +/− vs Revision +/−). The test of overall significance was performed through the c^2^ test, with values of *P* > 0.05 considered statistically significant. Besides, the Kaplan-Meier estimation plot was assessed, and the log-rank test was calculated to verify any significant association.

## Results

### Population

**Figure 1.**
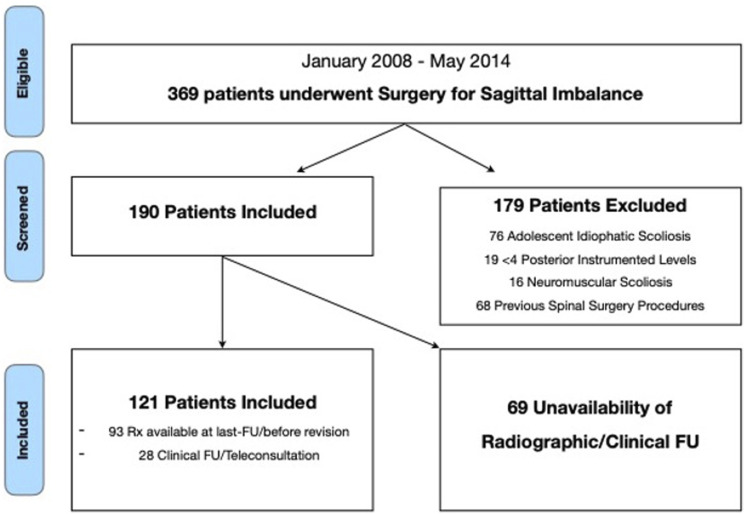
Flowchart.

Three hundred sixty-nine (369) patients were treated for ASD between 2008 and 2014. One hundred twenty-one (121) patients fulfilled the inclusion criteria and exclusion criteria and were definitively enrolled in the study (Figure 1). The pre-operative characteristics of the patients are presented in Table 1. Patients were classified as follow: 16 Global Kyphosis (GK); 58 Lumbar Kyphosis (LK); 2 Lower Lumbar Kyphosis (LLK); 10 Thoracic Kyphosis (TK); 35 Thoracolumbar Kyphosis (TLK).

**Table 1.** Pre-operative Characteristics of the Patients.

Characteristics	Mean ± SD
Mean age	70
Sex	• F: 103
	• M: 18
Lamartina-berjano classification	• GK: 16
	• LK: 58
	• LLK: 2
	• TK: 10
	• TLK: 35
Pelvic incidence (PI)	52 [±12]
Pelvic tilt (PT)	25 [±12]
Sacral slope (SS)	27 [±13]
Maximum lumbar lordosis (LL)	38 [±24]
L4-S1 lumbar lordosis (LLL)	30 [±16]
Thoracic kyphosis (TK)	38 [±120]
Sagittal vertical axis (SVA)	62 [±63]
Global tilt (GT)	32 [±19]

### Mechanical Complications

**Figure 2.**
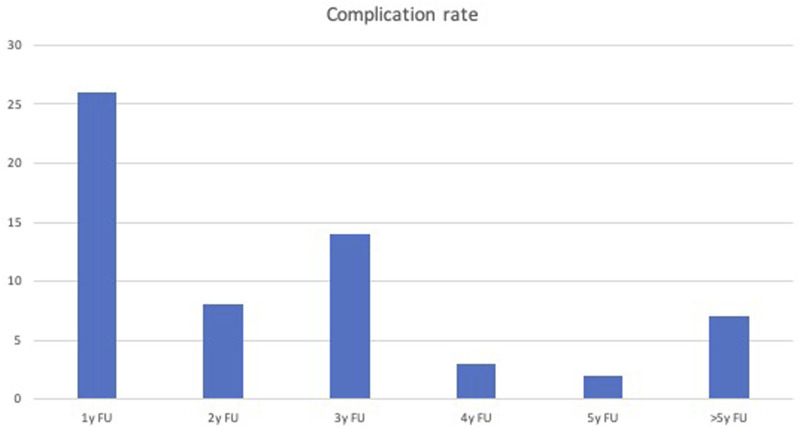
Pre-operative characteristics of the patients.

In our cohort, the revision surgery for clinically-relevant mechanical complication rate was 49,6% (60 out of 121 patients). In Figure 2 we reported the rates of complications divided per year along the follow-up. As shown in the figure, the risk of complications decreases at the fourth year after surgery, but is still present at a longer follow-up. The surgical indication followed the Lamartina-Berjano Classification criteria in 90 patients out of 121 (74%).

### Lamartina-Berjano Classification Validation

We divided the cohort in 4 different groups:

#### Group A

Patients with proper postoperative alignment (GAPscore ≤4) and the respect of Lamartina-Berjano Classification in the treatment (LB +)

#### Group B

Patients with proper postoperative alignment (GAPscore ≤4) without the respect of Lamartina-Berjano Classification in the treatment (LB -)

#### Group C

Patients without proper postoperative alignment (GAPscore >4) with the respect of Lamartina-Berjano Classification in the treatment (LB +)

#### Group D

Patients without proper postoperative alignment (GAPscore >4) without the respect of Lamartina-Berjano Classification in the treatment (LB -)

**Figure 3.**
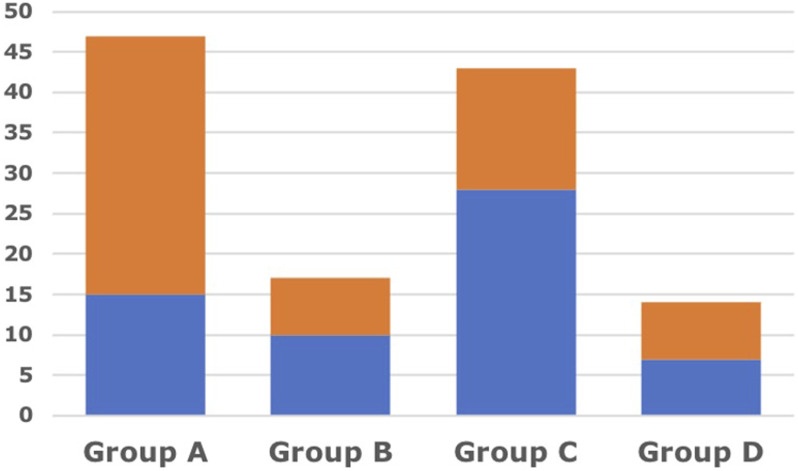
Rate of complications 32% (15/47) in Group A (GAPscore ≤ 4 + LB respected); 59% (10/17) in Group B (GAPscore ≤ 4 + LB non respected); 65% (28/43) in Group C (GAPscore > 4 + LB respected); 50% (7/14) in Group D (GAPscore > 4 + LB non respected).

We allocated 47 patients in Group A, 17 in Group B, 43 in Group C and 14 in Group D. We observed a complication rate of 32% in Group A (15/47), of 59% in Group B (10/17), of 65% in Group C (28/43) and of 50% in Group D (7/14) (Figure 3).

We performed a univariate analysis on the respect of Lamartina-Berjano Classification in the cohort of patients with postoperative GAPscore ≤4. We built a 2 × 2 table (Respected +/− vs Revision +/−) (Table 2). The chi-squared test showed a statistical association with a *P* = 0.05 - [c^2^ (2,N = 64) = 3,80].

**Table 2.** Chi-square Test.

	Mechanical complications	Non mechanical complications	Tot
L-B classification respected	15	32	47
L-B classification non respected	10	7	17
Tot	25	39	64

2x2 table (Respected +/- vs. Revision +/-); X^2^ (2, N = 64) = 3.80, *p* = 0.05.

**Figure 4.**
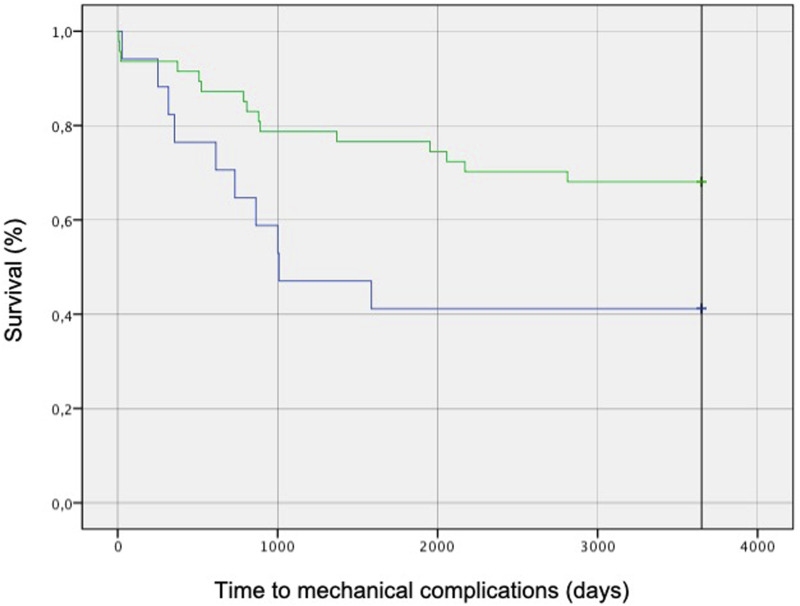
Survival curves Survival curves for patients with post-operative GAPscore ≤ 4 draw using Kaplan-Mayer method. Log rank test showed statistically significant differences between these curves (p = 0,03 - [c^2^ (N = 64) = 4,707].

Through the Kaplan-Meyer methods, we draw the survival curves for patients with post-operative GAPscore ≤4 in which the treatment had respected L-B classification and patients in which had not. (Figure 4) Log rank test showed statistically significant differences between these curves (*P* = 0,03 - [c^2^ (N = 64) = 4707].

## Discussion

Adult spinal deformity frequently affects perceived axial pain and social activity and quality of life of affected patients.^3^ The surgical treatment of these conditions often includes long spinal fusions with eventual osteotomies for a proper correction of the spinal alignment. These complex surgical procedures are burdened by a high rate of intraoperative complications, but also the late mechanical complications are frequent, affecting patients’ outcomes and surgical revision occurrence. It is demonstrated in literature how this population experience a rate of complications up to 70.7% of cases,^12^ either surgical or medical or mechanical,^13^ how this leads to re-admission of patients in 56% of patients^4^ and how the revision rate is above 10% in the first postoperative year and exceeds 20% in the second postoperative year^14,15^. In the last years scientific literature moved towards two main goals: a deeper understanding of spinal alignment, balance, and their implications; on the other side, the proposal of new surgical techniques and concepts to reduce the rate of complications, based on observation of previous results.

It is well accepted how clinical status of patients with adult scoliosis is related to sagittal balance and how realigning the spine to targets based on PI is the main goal of adult^16-18^ and how this can be obtained in different ways: only posterior procedures eventually combined with osteotomies, anterior approaches to the thoracolumbar spine, more or less^19,20^ or combined procedures.^21^ All this scientific production has the noble aim of reducing the rate of complications, better understanding the underlying spinal mechanisms and implementing surgical options. However, the rate of complication is still high, affecting a huge part of this population.

The classification on the spinal sagittal alignment published by Lamartina and Berjano in 2014 had a double aim: the proper description of the spinal deformity, giving a common language to physicians, and the proposal of a specific surgical treatment for each deformity group. This classification is based on two main evaluations: the location of the deformity and the identification of compensating mechanisms. This brought to the definition of seven different deformity groups, with a different proposed treatment for each of them: cervical kyphosis, thoracic hyperkyphosis, thoracolumbar kyphosis, lumbar kyphosis, lower lumbar kyphosis, global kyphosis, pelvic kyphosis. All of these deformities are usually associated to specific compensation mechanisms and, according to the classification, should be treated with different strategies.

In our retrospective observational study, we enclosed 121 patients that received a spinal fusion for the correction of adult spinal deformity with sagittal imbalance. We observed how the respect of the treatment indications provided by the LB classification leads to a statistically significant reduction in mechanical complications in properly aligned patients in a long-term follow-up scenario. The 10 years follow-up was selected to evaluate the long term effect of the surgical correction of adult deformities. However, we observed how the risk of mechanical complications is significantly reduced after 4 years from surgery, but is still present at a longer term follow-up.

## Conclusion

Based on our retrospective review of adult deformity patients, we have demonstrated how the accordance to the indications proposed in the LB classification brings to a reduction in mechanical complications in a long-term follow-up setting, when considering the thoracolumbar deformities.

We thus recommend adopting the Lamartina-Berjano classification in the preoperative planning of adult deformity patients as a guide for the proper treatment of this population, in order to reduce the risk of a patient readmission for a mechanical complication and a consequent reduction in his or her quality of life.

The Lamartina-Berjano classification has been proved as effective in deformities affecting the thoracolumbar spine. Further studies are needed to validate the classification in cervical and pelvic kyphosis.

## Data Availability

The datasets used and/or analyzed in the present study are available from the corresponding author upon reasonable request.*
